# Association between being African-American, serum urate levels and the risk of developing hyperuricemia: findings from the Coronary Artery Risk Development in Young Adults cohort

**DOI:** 10.1186/ar3552

**Published:** 2012-01-06

**Authors:** Angelo L Gaffo, David R Jacobs, Cora E Lewis, Ted R Mikuls, Kenneth G Saag

**Affiliations:** 1 Birmingham VA Medical Center 700 South 19th Street, Birmingham, AL 35233, USA; 2 Divisions of Rheumatology 172 Shelby Interdisciplinary Biomedical Research Building, 1825 University Boulevard, Birmingham, AL 35294-2182, USA; 3 Division of Epidemiology and Community Health, School of Public Health, University of Minnesota 1300 South Second Street, Suite 300, Minneapolis, MN 55454, USA; 4 Department of Nutrition, University of Oslo, P.O.Box 1046 Blindern, N-0316 Oslo, Norway; 5 Division of Preventive Medicine, Department of Medicine, 1530 3rd Avenue South, Medical Towers, Birmingham, AL 35294-4410, USA; 6 Section of Rheumatology and Immunology, The University of Nebraska Medical Center, 986270 Nebraska Medical Center, Omaha, NE 68198, USA

## Abstract

**Introduction:**

Findings that African-American race/ethnicity is associated with higher concentrations of serum urate have not been adjusted for possible confounding factors or have not explored this question as a primary outcome. We tested this hypothesis in a bi-racial cohort of younger African-American and white men and women.

**Methods:**

Data from 5,049 participants at the Coronary Artery Risk Development in Young Adults (CARDIA) cohort baseline (1985 to1986) and follow-up for up to 20 years of individuals without hyperuricemia (defined as a serum urate of 6.8 mg/dL or more) at baseline were utilized. We determined associations between race, serum urate and the development of hyperuricemia in sex-specific cross-sectional and longitudinal analyses. Confounding factors examined included: age at enrollment, body mass index, development of hypertension, glomerular filtration rate, medication use, diet and alcohol intake and menopausal symptoms in women.

**Results:**

Referent to whites, African-American men and women had significantly lower concentrations of serum urate at baseline. African-American men had an essentially equal risk of developing incident hyperuricemia during follow-up compared with white men (multivariable adjusted HR = 1.12 (0.88 to1.40)). African-American women developed a significantly increased risk of hyperuricemia when compared to white women (HR = 2.31 (1.34 to 3.99)).

**Conclusions:**

Young African-American men and women had lower concentrations of serum urate than whites. During longitudinal follow-up, African-American women had a significantly increased risk of developing hyperuricemia when compared with white women, a difference that was not observed in men. Differences in production of serum urate or a more rapid decline in fractional excretion of serum urate are potential, albeit still unproven, explanations for these findings in African-American women.

## Introduction

Increased levels of serum urate have been reported in association with hypertension, chronic kidney disease and obesity [1-3]. Hyperuricemia develops when the serum concentration of urate increases above 6.8 milligrams per deciliter [4] and is a necessary condition for the development of gout, which has also been associated with adverse cardiovascular outcomes [5]. Both gout and adverse cardiovascular outcomes have an increased prevalence among African-Americans [6-10]. Some environmental risk factors for hyperuricemia, such as increased fructose intake, are increased among African-Americans [11]. However, there are conflicting data regarding a primary association between being African-American and higher serum urate concentrations when compared to other races. Cross-sectional secondary analyses from the United States National Health and Nutrition Examination Survey (NHANES I) found that African-Americans had increased serum urate levels when compared with whites; however, this analysis did not account for confounding factors [12]. In contrast, a small South African study reported that African women had significantly lower levels of serum urate than white women [13]. More recently, investigators working with data from adolescents participating in the United States National Health and Nutrition Examination Survey (1999 to 2006) described that African-Americans with and without metabolic syndrome had lower serum urate levels than their white and Hispanic counterparts [14].

To address this question further, we utilized data from the unique Coronary Artery Risk Development in Young Adults (CARDIA) cohort. We examined the sex-specific associations between race/ethnicity, serum urate levels and the subsequent hazard of developing hyperuricemia.

## Materials and methods

### Study population

The CARDIA cohort was established in 1985 to 1986 with the main objective of investigating factors that contribute to the development of coronary heart disease in young adults [15]. Study design, recruitment of participants and the Institutional Review Boards' approvals with individual informed consent processes have been described in detail [16]. Institutional Review Boards at all the participating institutions: the University of Alabama at Birmingham (Birmingham, coordinating center, IRB registration 00000726), the University of Minnesota (Minneapolis), Northwestern University (Chicago), and Kaiser Permanente (Oakland) gave approval for the CARDIA study that is sponsored by the United States National Heart, Lung, and Blood Institute. All participants provided a written informed consent prior to enrollment. Briefly, 5,115 subjects 18 to 30 years of age were initially recruited in Birmingham, AL, Chicago, IL, Minneapolis, MN and Oakland, CA. The sample was selected so there would be approximately equal numbers of African-American and white women and men. Over the past 20 years, seven examinations of this cohort have been performed, with a majority of those enrolled participating in these follow-up examinations (ranging from 91% at year 2 to 72% at year 20) [17]. For the purpose of our analyses, only those individuals with serum urate measurements at the baseline exam were included (*n *= 5,049).

### Study variables

Information about race, sex and age was provided by self-report. Self-reported race was the independent variable of interest for all analyses. Serum urate levels, measured at baseline and at years 10, 15 and 20, were used to construct the dependent variables. At baseline, serum urate measurements were completed using the uricase method [9]. In years 10, 15 and 20 a modification of this method was used in which urate was oxidized by uricase to produce peroxide (measured at year 20 as part of the Young Adult Longitudinal Trends in Antioxidants (YALTA) ancillary study) [18]. To allow full comparability of urate measures from these time points with baseline urate levels and to conform to National Institute of Standards Standard Reference Materials, serum urate levels were recalibrated based on a re-run of frozen samples, such as for year 10 (recalibrated = measured *1.012 + 0.577), for year 15 (recalibrated = measured *0.952 + 0.866), and for year 20 (recalibrated = measured *1.022 + 0.440).

Covariates were selected based on their potential effect on serum urate levels and included: age at enrollment, body mass index (BMI); glomerular filtration rate estimated using the modification of diet in renal disease (MDRD) equation, that is based on creatinine measurements done by the nephelometry method and calibrated to national standards as described in previous CARDIA publications [19,20]; use of diuretics; use of antihypertensive medication; intake of beer, wine, liquor, meat, seafood and dairy; total protein; and self-report of onset of menopause for women.

### Data analyses

A cross-sectional analysis with information collected at the baseline examination was initially performed to estimate the multivariable-adjusted serum urate by sex and race group. Different models were fitted for men and women given well-described sex differences in serum urate levels, handling of serum urate and differential responses to factors affecting serum urate [21-23]. A multiple linear regression technique was used, and in order to identify the main sources of confounding, covariates were progressively added into the model. Initially, a model minimally-adjusted for age at enrollment was fitted. Secondly, a model was fitted, including age, body mass index, glomerular filtration rate, presence of hypertension, use of diuretics and use of other antihypertensive medication at baseline. Finally, the fully adjusted model included all the variables described, plus dietary variables (intake of meat, dairy, seafood and total protein), and alcohol intake information (intake of beer, wine and liquor) as these have been described to affect serum urate levels [22-24]. Race-specific urate levels were compared within gender using analysis of variance techniques with a significance level set at 0.05.

A longitudinal time-to-event hazard ratio with development of hyperuricemia (defined as a serum urate of 6.8 milligram per deciliter or more), which represents the solubility threshold of serum urate [4] or loss to follow-up as censoring events, was performed. Individuals with hyperuricemia at baseline were excluded from this analysis, lowering the analytic sample to 4,348 individuals. In this analysis, time-varying covariates (where baseline values are replaced by the follow-up values of the covariates when the participant reaches different points during follow-up) were progressively added into the model. Initially, a model minimally-adjusted for age at enrollment was fitted. Secondly, a model including age, body-mass index, glomerular filtration rate, presence of hypertension, use of diuretics and use of antihypertensive medications was fitted. A subsequent model included all the variables described plus dietary and alcohol intake information. For women, a third model included the onset of menopause. The fully adjusted model was completed with the addition of the baseline serum urate. Whites were the reference group against which African-Americans were compared within each sex stratum. SAS 9.2 (Cary, NC, USA) was used for all statistical analyses.

## Results

### Characteristics of study population

Table 1 shows the sex- and race-specific characteristics of the samples used in the cross sectional and longitudinal analyses. At baseline, African-American men had comparable BMI to their white counterparts. In contrast, African-American women had higher BMI than whites. Glomerular filtration rates were greater for African-American men and women than for whites. A greater proportion of African-Americans had hypertension and used diuretic medications when compared with whites. On the other hand, more white men reported taking medications for hypertension than African-American men. Only 11 individuals reported use of medicines to treat gout at some point in the study. For dietary variables, African-Americans had greater reported intakes of meat and total protein than whites (Table 1).

**Table 1:** Characteristics of the baseline cross-sectional (CS) and longitudinal (L) analysis populations at baseline

	Cross sectional analysis population				Longitudinal analysis population			
	**Men**		**Women**		**Men**		**Women**	
	**AA** **(*n *= 1,143)**	**W** **(*n *= 1,161)**	**AA** **(*n *= 1,447)**	**W** **(*n *= 1,298)**	**AA** **(*n *= 849)**	**W** **(*n *= 812)**	**AA** **(*n *= 1,409)**	**W** **(*n *= 1,278)**
Age *	24.2 (3.8)	25.4 (3.4)	24.4 (3.9)	25.4 (3.4)	24.0 (3.7)	25.4 (3.3)	24.3 (3.8)	25.4 (3.4)
**BMI**	24.5 (4.3)	24.3 (3.6)	25.8 (6.5)	23.1 (4.4)	23.8 (3.6)	23.7 (3.0)	25.7 (6.4)	23.0 (4.3)
**GFR†**	99.2 (17.7)	86.6 (15.0)	98.5 (19.3)	81.7 (14.9)	101.1 (17.6)	88.3 (15.1)	98.9 (19.2)	81.9 (14.8)
**Hypertension‡ (%)**	4.0	2.8	1.9	0.7	3.2	2.1	1.8	0.6
**Antihypertensive use§¶ (%)**	0.4	1.4	1.2	1.4	0	1.4	1.3	1.0
**Diuretic use (%)**	0.7	0.5	1.6	1.0	0.2	0.4	1.4	0.9

	**Cross sectional analysis population**				**Longitudinal analysis population**			

	**Men**		**Women**		**Men**		**Women**	
	AA (*n *= 1,143)	W (*n *= 1,161)	AA (*n *= 1,447)	W (*n *= 1,298)	AA (*n *= 849)	W (*n *= 812)	AA (*n *= 1,409)	W (*n *= 1,278)
**Beer intake||**	2 (0 to 6.0)	2 (0 to 7.0)	0 (0 to 0)	0 (0 to 2.0)	1 (0 to 6.0)	2 (0 to 6.0)	0 (0 to 0)	0 (0 to 2.0)
**Liquor intake||**	0 (0 to 0)	0 (0 to 1.0)	0 (0 to 0)	0 (0 to 2.0)	0 (0 to 0)	0 (0 to 1.0)	0 (0 to 0)	0 (0 to 2.0)
**Wine intake||**	0 (0 to 0)	0 (0 to 1.0)	0 (0 to 0)	0 (0 to 2.0)	0 (0 to 0)	0 (0 to 1.0)	0 (0 to 0)	0 (0 to 2.0)
**Meat intake ¶**	6.4 (4.9)	3.9 (2.7)	3.8 (2.8)	2.0 (1.6)	6.3 (5.0)	3.7 (2.6)	3.7 (2.7)	2.0 (1.6)
**Seafood intake¶**	0.8 (1.2)	0.6 (0.8)	0.6 (1.1)	0.5 (0.6)	0.7 (1.2)	0.6 (0.8)	0.6 (1.1)	0.5 (0.6)
**Dairy intake¶**	6.1 (7.1)	5.8 (5.0)	3.7 (3.8)	4.1 (3.1)	6.1 (6.1)	6.0 (5.0)	3.7 (3.8)	4.1 (3.1)
Protein intake**	144 (88)	126 (56)	91 (49)	81 (33)	145 (92)	125 (55)	91 (49)	81 (34)

AA, African-American; W, White; BMI, body-mass index in kilograms per square meter; GFR, glomerular filtration rate *All values expressed as mean (standard deviation) unless noted. † In milliliters per minute. Values obtained using the Modification of Diet in Renal Disease (MDRD) equation [20]. ‡ Defined as a systolic blood pressure ≥ 140 or a diastolic blood pressure ≥ 90 millimeters of mercury. § Excluding diuretics.|| Alcohol intake values expressed as median (inter-quartile range) given skewed distribution of data points. Measured in servings/week, where one serving of beer is equal to a 12-ounce bottle, one serving of liquor is equal to a 1.5-ounce shot, and one serving of wine is equal to a 5-ounce glass

¶ Reported as servings/day (meat, seafood, dairy) or grams/day (protein). One meat or seafood serving is equal to one ounce. One serving of diary varies according to the composition: for milk a serving is equal to one cup or eight fluid ounces. For cheese a serving is two ounces for processed, two cups for cottage and three cups for dry curd. For yogurt is it is one cup and for ice cream it is one-half cup. ** In grams per day.

African-American women had greater rates of menopause onset by year 20 of follow-up. Hyperuricemia at baseline and development of hyperuricemia was more frequent for African-American women (2.4% at baseline and 10% cumulative by year 20) when compared with whites (1.9% at baseline and 3.9% cumulative at year 20), but the opposite was observed for men (25.6% at baseline for African-Americans versus 29.7% for whites and 29.2% cumulative at year 20 for African-Americans versus 34.0% for whites).

### Baseline serum urate estimations

Table 2 shows the multivariable estimations of serum urate by race and sex category and the mean serum urate differences between African-Americans and whites at baseline. For both men and women, African-Americans had lower levels of serum urate. These differences were statistically significant even after adjustment for potential confounders, such as BMI, glomerular filtration rate, medication use. The differences between African-Americans and whites were significant in the final model that included dietary variables and alcohol intake.

**Table 2:** Multivariable-adjusted serum urate at baseline

	African-American men	White men	Mean difference (95% CI)	African-American women	White women	Mean difference (95% CI)
Age-adjusted*	6.06	6.28	0.22 (0.10 to 0.34)	4.41	4.53	0.11 (0.01 to 0.22)
**Model 1†**	6.59	6.70	0.11 (0.00 to 0.22)	4.85	4.97	0.12 (0.00 to 0.22)
**Model 2‡**	6.35	6.51	0.16 (0.04 to 0.27)	4.70	4.86	0.16 (0.05 to 0.27)

*All values expressed in milligrams per deciliter. †Adjusted for age at inception, body-mass index, glomerular filtration rate, presence of hypertension, use of diuretics at baseline, use of other antihypertensive medication at baseline. ‡ Adjusted for variables described for model 1 plus intake of beer, wine, liquor, meat, seafood, dairy, total protein

### Risk of developing hyperuricemia

Among men without hyperuricemia at baseline, a total of 15.4%, 23.9% and 31.6% first developed hyperuricemia by years 10, 15 and 20, respectively. For women without hyperuricemia at baseline, the first development of hyperuricemia at those years was of 1.9%, 4.2% and 7.1% respectively. Table 3 shows the risk of developing hyperuricemia in African-Americans when compared with their white counterparts. On fully adjusted multivariable analysis, findings differed for men and women, with African-American men having a similar hazard (HR = 1.12; 95% CI 0.88 to 1.40) and women an increased hazard (HR = 2.31; 95% CI 1.34 to 3.99) of developing hyperuricemia when compared to whites. The fully adjusted hazard of developing hyperuricemia increased progressively in women. At year 10, the HR (95% CI) was 0.88 (0.28 to 2.98) and at year 15 was 1.84 (0.87 to 3.92). In men, the hazard of developing hyperuricemia remained constant. At year 10, the HR was 1.23 (0.90 to 1.69) and at year 15 it was 1.26 (0.98 to 1.62).

**Table 3:** Hazard ratio of developing hyperuricemia* in African-Americans compared to whites, stratified by sex

	Men		Women	
	**Hazard ratio**	**95% Confidence interval**	**Hazard ratio**	**95% confidence interval**
**Age-adjusted**	0.87	0.73 to 1.04	2.79	2.02 to 3.86
**Model 1 †**	1.14	0.95 to 1.36	2.36	1.67 to 3.35
**Model 2 ‡**	0.94	0.75 to 1.17	1.92	1.23 to 3.00
**Model 3 §**	Not applicable		1.94	1.14 to 3.29
**Model 4 ||**	1.12	0.88 to 1.40	2.31	1.34 to 3.99

*Defined as the finding of a serum urate of 6.8 mg/deciliter or higher at any follow-up visit (years 10, 15, or 20). † Adjusted for age at enrollment, baseline body-mass index, glomerular filtration rate, presence of hypertension, use of diuretics, and use of other antihypertensive medication. ‡ Adjusted for variables described for model 1 plus baseline intakes of beer, wine, liquor, meat, seafood, dairy, total protein. § Adjusted for variables described in model 2 plus onset of menopause by then end of follow-up. || Adjusted for variable described in model 2 (men) and model 3 (women) plus baseline serum urate levels

## Discussion

We report lower serum urate concentrations for young African-American men and women (mean population age was 24 years old) when compared to whites. For African-American men without hyperuricemia at baseline, the risk of developing hyperuricemia after 20 years of follow-up was non-significantly different than for their white counterparts, but for women without hyperuricemia at baseline this risk reverses and African-Americans have a 2.3-fold increased risk of developing hyperuricemia when compared with whites.

Our cross-sectional analysis results show a difference between African-American and white women and men of approximately 0.16 mg/dL. An initial conclusion from this analysis is that young African-Americans are not likely to have higher serum urate than young individuals from other races or ethnicities and this makes it unlikely that there is a genetic association with hyperuricemia. The magnitude of this difference is small but comparable to the increase in serum urate associated with one additional serving of alcohol per day at this young age [25]. However, it is unlikely to be clinically relevant for gout or cardiovascular conditions in this population. Our results are similar to the ones recently published after analyzing a population of adolescents (ages 13 to 19) from the NHANES survey (1996 to 2000) where whites with and without metabolic syndrome were found to have higher serum urate than Hispanics and African-Americans [14]. The Profiles of Obese Women with the Insulin Resistance Syndrome (POWIRS) study in South Africa compared African women with whites at a mean age of 31 years. Despite having higher blood pressure levels, and after adjustment for body-mass index, African women had lower serum urate levels than whites [13]. Despite the small numbers and ethnic differences between African-Americans and South Africans, the age ranges and general conclusion of this study are concordant with our baseline cross-sectional result for women.

Our longitudinal analysis results are similar to those reported by Fang and Alderman, where they examined serum urate using data from the National Health and Nutrition Examination Survey (NHANES I) in a slightly older population [12]. Consistent with our longitudinal analysis findings, they found that African-Americans had higher levels of urate than whites (5.7 versus 5.5 milligrams per deciliter) in a population with a slight predominance of women. However, they did not stratify by sex and they did not adjust this analysis for potential confounders.

Potential environmental factors and genetic and physiologic mechanisms explaining these differences in the development of hyperuricemia between African-Americans and whites remain obscure. Urate-excretion abnormalities are the most common causes of hyperuricemia. African-Americans have been previously reported to have similar renal filtration rates and lower renal perfusion than whites [26], although we observed a higher filtration rate for African-Americans in this cohort. However, the associations between race/ethnicity and serum urate we observed persisted even after adjustment for glomerular filtration rate, which could be a poor representation of fractional excretion of urate. The fractional excretion of serum urate has been reported to be similar among elderly African-Americans and whites in a genetic association study [27]. There is no information, to our knowledge, about fractional excretion of urate in young African-Americans, and it could be postulated that a higher fractional excretion of urate protects young African-Americans from developing hyperuricemia until they develop kidney dysfunction and overall lower rates of glomerular filtration later in life. Also, genetic differences in urate handling by the renal tubules have been described in association with the SLC2A9 gene [28,29] and others (ABCG2*, NPT1*) [30]. Similar to previous reports in whites, *SLC2A9 *has been found to be significantly associated with serum urate concentrations in population-based samples of African Americans [31]. Interestingly, this effect was found to be stronger in females than in males. Other genetic associations have not been investigated in African-Americans. Within the CARDIA cohort, Rathmann and colleagues previously identified changes in BMI that are closely associated with changes in serum urate among African-American men and white men and women [32]. Of note, our general conclusions were not different after adjustment for body-mass index. It is also possible that development of hyperuricemia among African-American men was not captured because their mean times of follow-up were lower than for whites (14.9 versus 17.4 years), with larger proportions of the former missing the serum urate collections at years 15 and 20. We studied the pattern of the increase in serum urate in those individuals with missing exams and it did not differ from those without missing exams.

The strengths of our analyses include: the unique population studied that was well balanced in proportions of African-American and white men and women, the long duration of mean follow-up for the longitudinal analysis, and a thorough assessment of covariates. Limitations included: the subjectivity of the exposure variable (self-defined race plus substantial racial admixture among the African-Americans), ascertainment of longitudinal outcomes at fixed time points (years 10, 15, and 20 of follow-up), a lower mean time of follow-up for African-American men, inability to include fructose intake information as a covariate, and the possibility of residual confounding. It is also possible that adjusting for a more specific measure of urate filtration and excretion would have provided more precise estimates.

## Conclusions

We describe an independent association between being African-American and lower levels of serum urate when compared with whites, in a population of young adults. After 20 years of follow-up, women developed a greater risk of developing hyperuricemia when compared to whites. For African-American men within the age ranges we studied, the risk of developing hyperuricemia over time equaled the risk in white men. In African-American women, residual environmental and dietary factors along with genetics may play a role for increases in serum urate later in life. Future areas for research that should follow this study include: confirmation of our findings in larger epidemiological datasets, follow-up of older adults to confirm if African-American men developed higher rates of hyperuricemia later in life explaining a higher gout prevalence, studies exploring racial genetic differences in body handling of serum urate, and physiological studies to confirm whether different rates of renal filtration or excretion of serum urate are present in African-Americans compared to other races/ethnicities.

## Abbreviations

BMI: body mass index; CARDIA: Coronary Artery Development in Young Adults study; HR: Hazard ratio; MDRD: modification of diet in renal disease; NHANES I: National Health and Nutrition Examination Survey; POWIRS: Profiles of Obese Women with the Insulin Resistance Syndrome study; YALTA: Young Adult Longitudinal Trends in Antioxidants study.

## Competing interests

Dr. Saag discloses having received research funding from Takeda Pharmaceuticals and being a consultant for Ardea Pharmaceuticals, URL Pharma and Novartis. All other authors report no conflict of interest.

## Authors' contributions

ALG, DRJ and KGS were involved in the conception and design of the study, the analysis and interpretation of data, and in manuscript preparation. CEL participated in the conception and design of the study, data acquisition, and manuscript preparation. TRM was involved in analysis and interpretation of data, and in manuscript preparation. All authors and the CARDIA cohort steering committee read and approved the final manuscript.
